# Expansion of B-1a Cells with Germline Heavy Chain Sequence in Lupus Mice

**DOI:** 10.3389/fimmu.2016.00108

**Published:** 2016-03-24

**Authors:** Nichol E. Holodick, Leilani Zeumer, Thomas L. Rothstein, Laurence Morel

**Affiliations:** ^1^Center for Oncology and Cell Biology, The Feinstein Institute for Medical Research, Manhasset, NY, USA; ^2^Department of Pathology, Immunology, and Laboratory Medicine, University of Florida, Gainesville, FL, USA; ^3^Department of Medicine, The Hofstra Northwell School of Medicine, Manhasset, NY, USA; ^4^Department of Molecular Medicine, The Hofstra Northwell School of Medicine, Manhasset, NY, USA

**Keywords:** B cells, B-1 cells, autoimmunity, lupus erythematosus, systemic, repertoire analysis, mouse model, natural antibodies

## Abstract

B6.*Sle1.Sle2.Sle3* (B6.TC) lupus-prone mice carrying the NZB allele of *Cdkn2c*, encoding for the cyclin-dependent kinase inhibitor P18^INK4^, accumulate B-1a cells due to a higher rate of proliferative self-renewal. However, it is unclear whether this affects primarily early-appearing B-1a cells of fetal origin or later-appearing B-1a cells that emerge from bone marrow. B-1a cells are the major source of natural autoantibodies, and it has been shown that their protective nature is associated with a germline-like sequence, which is characterized by few N-nucleotide insertions and a repertoire skewed toward rearrangements predominated during fetal life, V_H_11 and V_H_12. To determine the nature of B-1a cells expanded in B6.TC mice, we amplified immunoglobulin genes by PCR from single cells in mice. Sequencing showed a significantly higher proportion of B-1a cell antibodies that display fewer N-additions in B6.TC mice than in B6 control mice. Following this lower number of N-insertions within the CDR-H3 region, the B6.TC B-1a cells display shorter CDR-H3 length than B6 B-1a cells. The absence of N-additions is a surrogate for fetal origin, as TdT expression starts after birth in mice. Therefore, our results suggest that the B-1a cell population is not only expanded in autoimmune B6.TC mice but also qualitatively different with the majority of cells from fetal origin. Accordingly, our sequencing results also demonstrated the overuse of V_H_11 and V_H_12 in autoimmune B6.TC mice as compared to B6 controls. These results suggest that the development of lupus autoantibodies in these mice is coupled with skewing of the B-1a cell repertoire and possible retention of protective natural antibodies.

## Introduction

Murine B-1a cells are a unique B-lymphocyte lineage characterized by phenotypic, functional, and ontologic characteristics (1, 2). B-1a cells are defined by surface marker expression of IgM^hi^IgD^lo^CD45R^lo^CD5^+^CD43^+^CD19^hi^ and are found in the peritoneal cavity, spleen, and bone marrow (3, 4). Functionally, B-1a cells exhibit unique signaling characteristics (4–6), are potent antigen-presenting cells (7), and spontaneously produce 80–90% of natural serum IgM in mice (8). Natural IgM is non-immune, low-affinity immunoglobulin (Ig) that is both polyreactive and autoreactive. It functions in infection, atherosclerosis, B cell homeostasis, inflammation, and autoimmunity [reviewed in Ref. (9)]. Minimal N-region addition contributes to the germline-like nature of natural IgM. Furthermore, natural IgM manifests biased variable heavy chain (VH) gene usage in favor of V_H_11 and V_H_12, which are specific for phosphatidylcholine (PtC), a major component of cell membrane phospholipids (3, 10–13). This unique germline structure of natural IgM is established during the early fetal and neonatal development of B-1a cells (8).

The polyreactive nature of natural IgM provides initial defense against both bacterial and viral pathogens, which affords the organism protection during the period preceding generation of high-affinity antigen-specific antibodies produced by germinal center B-2 cells (3, 4, 14–17). The autoreactive quality of natural IgM has been shown to aid in the elimination of excess autoantigens through the removal of apoptotic cells and noxious molecular debris, thereby maintaining homeostasis and preventing inflammation (9, 18). These autoreactive natural antibodies are often directed against cell membrane components, such as PtC and phosphorylcholine (PC), which is the polar head group of PtC and is a major microbial cell wall determinant (19). Interestingly, such components are closely related to those also present on pathogens, which suggest that the natural autoreactive repertoire also react with common pathogens (20).

While this cross-reactivity of natural autoreactive antibodies with pathogens is beneficial, it highlights the importance of regulating B-1a cell expansion. Accumulation of B-1a cells has been shown in the NZM2410 (NZB × NZW F_1_ hybrid) mouse model of systemic lupus erythematosus (SLE) (21, 22). However, the role of B-1a cells in lupus is still unclear (23). Some studies have demonstrated a role for B-1a cells *via* production of IL-10 (22), increase in antigen presentation (22), or with overexpression of osteopontin, resulting in expansion of B-1a cells and increased anti-dsDNA antibody production (24). In other models, however, B-1a cells do not contribute to disease (25, 26).

The expansion of B-1a cells in the NZM2410 model was traced to the *Sle2c1* lupus susceptibility locus, which contains *Ckdn2c* (27, 28). The *Ckdn2c* gene encodes for p18^INK4c^, which is a cyclin-dependent kinase inhibitor that controls progression through G1 of the cell cycle (28). These studies demonstrated that the expansion of B-1a cells is intrinsic. B6.*Sle2c1* B-1a cells showed increased proliferation at rest, as well as increased resistance to cell death. In addition, B-1a cell reconstitution from fetal liver, adult bone marrow, and adult spleen was higher in B6.*Sle2c1* than in control C57/BL6 (B6) mice following lethal irradiation (21). Furthermore, comparison of p18^−/−^ mice with B6.*Sle2c1* mice demonstrated that both produced autoantibodies; however, the amount produced by p18^−/−^ mice was greater. This demonstrates that the control of the B-1a cell population depends on the amount of p18. B6.*Sle2c1* mouse B cells have fourfold less *Ckdn2c* than normal mice, whereas p18^−/−^ mice completely lack *Ckdn2c* (28). Together, these results demonstrate an important role for p18 in B-1a cell numbers, which in turn affects the production of autoantibodies and development of autoimmunity. However, the origin of B-1a cell expansion in B6.TC, B6.Slec1, and p18^−/−^ mice could be due to an increase in proliferation of early-appearing fetal-derived B-1a cells or heightened production of later-appearing bone marrow-derived B-1a cells. As the repertoires of early- and later-appearing B-1a cells differ, these two possibilities can be distinguished. Herein, we investigated whether significant changes to the natural IgM repertoire occur in triple congenic B6.*Sle1.Sle2.Sle3* (B6.TC) lupus-prone mice. These mice carry the *Sle2c1* locus that drives B-1a cell expansion and present clinical autoimmune pathology that has been described for the NZM2410 pathology (29). B6.TC mice carry the NZM2410 susceptibility loci on a B6 genetic background (>95%) that includes both heavy and light immunoglobulin chains, which allow to directly compare the lupus-prone B6.TC mice to the control B6 mice. Specifically, we found that the expansion of B-1a cells in B6.TC mice is associated with repertoire skewing toward V_H_11 and V_H_12 usage.

## Materials and Methods

### Mice

B6.NZM-*Sle1*^NZM2410/Aeg^*Sle2*^NZM2410/Aeg^*Sle3*^NZM2410/Aeg^/LmoJ (B6.TC) congenic mice have been previously described (29). B6.TC lupus-prone and C57BL6/J (B6) control mice were cared for and handled in accordance with National Institutes of Health and institutional guidelines.

### Single-Cell Sequencing and Analysis

Peritoneal washout cells were obtained from 8-week-old wild-type B6 mice and 8-week-old B6.TC mice (two each). The cells were stained with fluorescence-labeled antibodies to CD45R/B220 (clone RA3-6B2), CD5 (clone 53-7.3), and CD23 (clone B3B4) (BD Biosciences). B-1a cells (CD45R^lo^/CD5^+^/CD23^−^) were then purified using an Influx cell sorter (BD Biosciences). Post-sort reanalysis of B cell populations showed them to be ≥98% pure. Peritoneal B-1a cells were sorted into a 96-well plate containing 20 μl of lysis buffer per well (dH_2_O, RNase Out, 5× SuperScript III Buffer, DTT, IgePAL, and Carrier RNA). Reverse transcription was performed (42°C – 10 min, 25°C – 10 min, 50°C – 60 min, 94°C – 5 min, hold at 4°C) after addition of random hexamers, dNTP mix, and SuperScript III reverse transcriptase. Semi-nested PCR was performed using the cDNA diluted 1:1. Using Qiagen’s HotStart Taq Plus, 2.5 μl of cDNA was used for the first PCR reaction with previously described primers (30). The product from this first PCR reaction was then diluted 1:100 and 2 μl of the diluted product was added to the second PCR reaction. The products were purified and then sequenced (Genewiz) using the forward primer. Sequences were then analyzed using an online sequence analysis tool for VDJ sequences (IMGT, the international ImMunoGeneTics information system).

### Statistics

Comparisons were conducted between the pooled B6.TC and B6 sequences and the two strains using Graphpad Prism 6.0 with two-tailed tests, as indicated in the figure legends.

## Results

### Lupus-Prone Triple Congenic Mice Display an Increase in Duplicate Sequences

**Figure 1:**
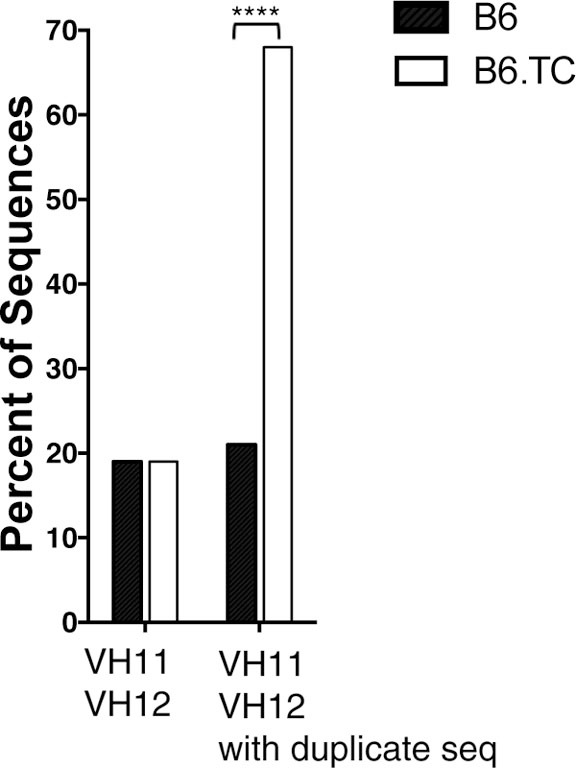
**Percent of V_H_11 and V_H_12 representation**. Peritoneal B-1a cells were single-cell sorted from 8-week-old B6 control mice and B6.TC lupus-prone mice. IgM was amplified and sequenced as detailed in the Section “Materials and Methods.” The percent of sequences that utilized V_H_11 and V_H_12 are shown for all unique sequences (left, B6 *n* = 62; B6.TC *n* = 47), and all sequences obtained, including the duplicates (right, B6 *n* = 105; B6.TC *n* = 146). Statistical analysis was performed using chi-square analysis (2 × 2 using the VH of interest and all others as the two categories), VH11/VH12 with duplicate sequences ****p < 0.0001.

The B-1a cell repertoire of B6.TC mice was compared to control B6 mice. Repertoire analysis was performed by PCR amplification of the V_H_DJ_H_ region from individual peritoneal B-1a cells, which were previously shown to accumulate in B6.TC mice (21, 28). Interestingly, the B-1a cells from B6.TC mice had a significantly larger number of IgM sequences with identical V_H_, D_H_, and J_H_ segments as well as identical CDR3 regions than B6 mice (Table 1, B6.TC: 108 out of 146 total sequences; B6: 50 out of 105 total sequences; *p* = 0.0335, Mann–Whitney test). As stated in previously published work (30), it cannot be determined whether these sequences containing identical V_H_, D_H_, J_H_, and CDR3 regions result from a single clonal expansion or from analysis of independent cells with identical rearrangements. Therefore, we will refer to such sequences as duplicate sequences instead of clones. Furthermore, V_H_ usage within the duplicate sequences differed significantly between B6 and B6.TC mice. As shown in Table 1, the duplicate sequences with the highest frequency in B6 mice utilized V_H_1–55 (58%), whereas the most frequent duplicate sequences in B6.TC mice utilized V_H_11 and V_H_12 (43 and 46%, respectively). While both V_H_11 and V_H_12 utilization (20 and 8%, respectively) was observed in B6 mice, this percentage of duplicate sequences was significantly less than that seen in B6.TC mice (Figure 1). These results suggest that the accumulation of peritoneal B-1a cells seen in B6.TC mice might be influenced by autoantigen since there is an expansion of B-1a cells utilizing V_H_11 and V_H_12, which are specific for PtC, a major component of cell membrane phospholipids.

**Table 1:** **Duplicate sequences**.

Sample	CDR3 AA sequence	VH	DH	JH	Number of duplicate sequences	Percent of duplicates
B6	AGDSHGYWYFDV	IGHV12-3*01	IGHD1-1*02	IGHJ1*03	2	
	AGDVTGYWYFDV	IGHV12-3*01	IGHD4-1*01	IGHJ1*02	2	
	ARFYYYGSSYAMDY	IGHV1-55*01	IGHD1-1*01	IGHJ4*01	7	
	ARRDYGSSYWYFDV	IGHV1-55*01	IGHD1-1*01	IGHJ1*02	22	
	ARHYYGSSYYFDY	IGHV5-6*01	IGHD1-1*01	IGHJ2*01	4	
	MRYGNYWYFDV	IGHV11-2*01	IGHD2-1*01	IGHJ1*03	7	
	MRYSNYWYFDV	IGHV11-2*01	IGHD2-5*01	IGHJ1*03	3	
	TREDYYYYGSSYYAMDY	IGHV5-9-1*02	IGHD1-1*01	IGHJ4*01	3	
					50	48
B6.TC	AGDNDGYWYFDV	IGHV12-3*01	IGHD2-3*01	IGHJ1*03	3	
	AGDNDGYYGFAY	IGHV12-3*01	IGHD2-3*01	IGHJ3*01	2	
	AGDYDGYWYFDV	IGHV12-3*01	IGHD2-3*01	IGHJ1*03	37	
	AGDYYGYWYFDV	IGHV12-3*01	IGHD1-1*02	IGHJ1*03	4	
	ARDYYGSSHYFDY	IGHV1-82*01	IGHD1-1*01	IGHJ2*01	2	
	ARELIYYGNYGYFDV	IGHV1-72*01	IGHD2-1*01	IGHJ1*03	2	
	ARPYYSNYYAMDY	IGHV2-9-1*01	IGHD2-5*01	IGHJ4*01	2	
	ARYYYGSSYAMDY	IGHV7-3*01	IGHD1-1*01	IGHJ4*01	2	
	MRYGNYWYFDV	IGHV11-2*01	IGHD2-1*01	IGHJ1*03	39	
	MRYGSSYWYFDV	IGHV11-2*01	IGHD1-1*01	IGHJ1*03	3	
	MRYSNYWYFDV	IGHV11-2*01	IGHD2-5*01	IGHJ1*03	8	
	TRTSGYFDY	IGHV6-6*01	IGHD1-3*01	IGHJ2*01	2	
	VRHYGSSYFDY	IGHV10-1*01	IGHD1-1*01	IGHJ2*01	2	
					108	74

*Peritoneal B-1a cells were single-cell sorted from 8-week-old C57BL/6 mice (B6) and B6.Sle1.Sle2.Sle3 (triple congenic, B6.TC) lupus-prone mice. IgM was amplified and sequenced as detailed in the Section “Materials and Methods.” Sequencing analysis revealed a number of sequences with identical CDR-H3 regions, which we refer to as duplicate sequences*.

### V_H_–D_H_–J_H_ Usage Shows Differences between B6 and B6.TC Mouse Repertoires

For analysis of V_H_, D_H_, and J_H_ usage, we evaluated the repertoire in two ways. First, we analyzed only sequences with unique CDR-H3 regions by removing all duplicate sequences. In the second method, we analyzed all sequences, which included the duplicate sequences.

**Figure 2:**
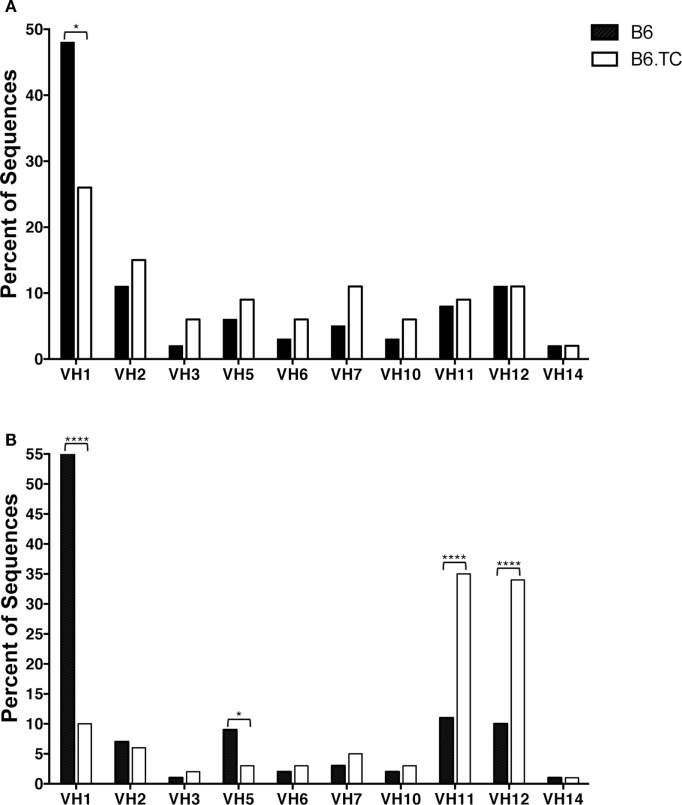
**V_H_ analysis of IgM from C57BL/6 and triple congenic peritoneal B-1a cells**. Immunoglobulins were amplified by PCR from single-cell sorted peritoneal B-1a cells obtained from B6 and B6.TC mice and evaluated for the variable (V) segment heavy chain usage. The percent of cells (sequences) expressing the V segment usage is displayed. Chi-squared test was used to determine significance. **(A)** Analysis of sequences with only unique CDR-H3 regions (B6, *n* = 62; B6.TC *n* = 47). **(B)** Analysis of all sequences obtained, including the duplicate sequences (B6, *n* = 105; B6.TC *n* = 146).

When analyzing only sequences with unique CDR-H3 regions, we found overall similarity in D_H_ and J_H_ usage with only one major significant difference in V_H_ usage. Among V_H_ gene segments, V_H_1 was expressed significantly less frequently by B6.TC B-1a cells (26%) as compared to B6 B-1a cells (48%) (*p* = 0.0034, chi-square test) (Figure 2A).

**Figure 3:**
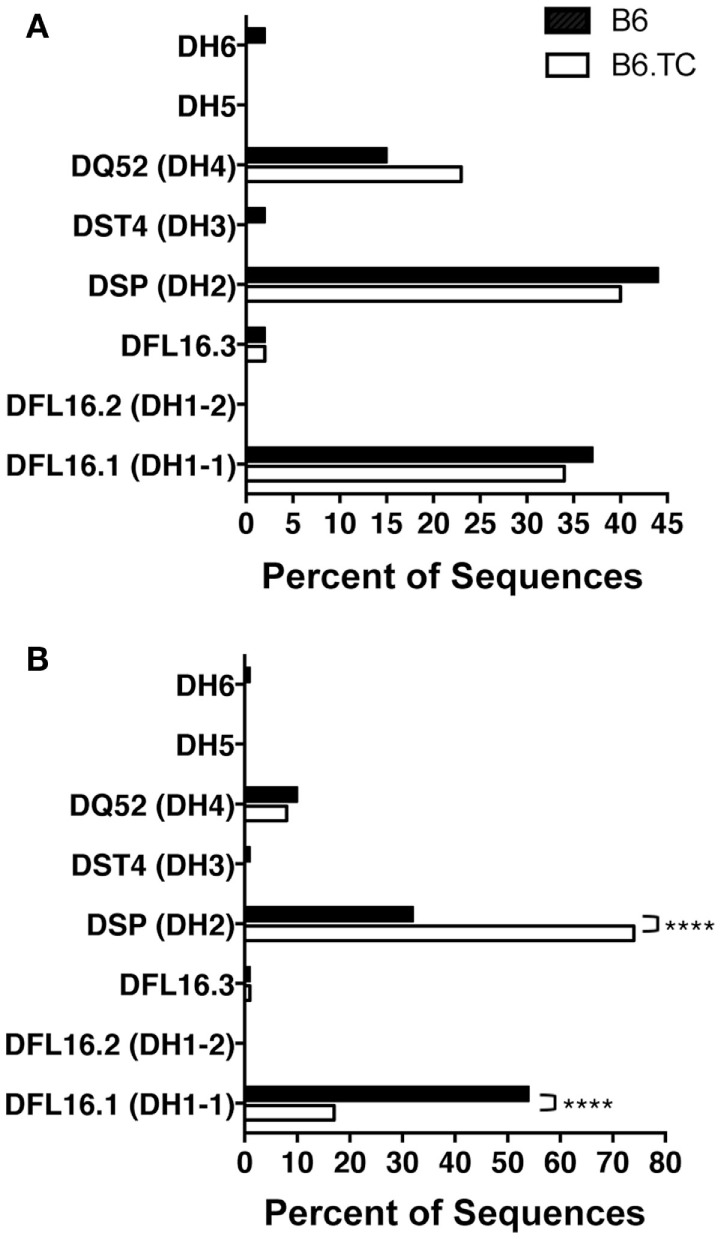
**D_H_ analysis of IgM from C57BL/6 and triple congenic mouse peritoneal B-1a cells**. Immunoglobulins were amplified by PCR from single-cell sorted peritoneal B-1a cells obtained from B6 and B6.TC mice and evaluated for the diversity (D) segment heavy chain usage. The percent of cells (sequences) expressing the D segment usage is displayed. Chi-squared test was used to determine significance. **(A)** Analysis of sequences with only unique CDR-H3 regions (B6, *n* = 62; B6.TC *n* = 47). **(B)** Analysis of all sequences obtained, including the duplicate sequences (B6, *n* = 105; B6.TC *n* = 146).

**Figure 4:**
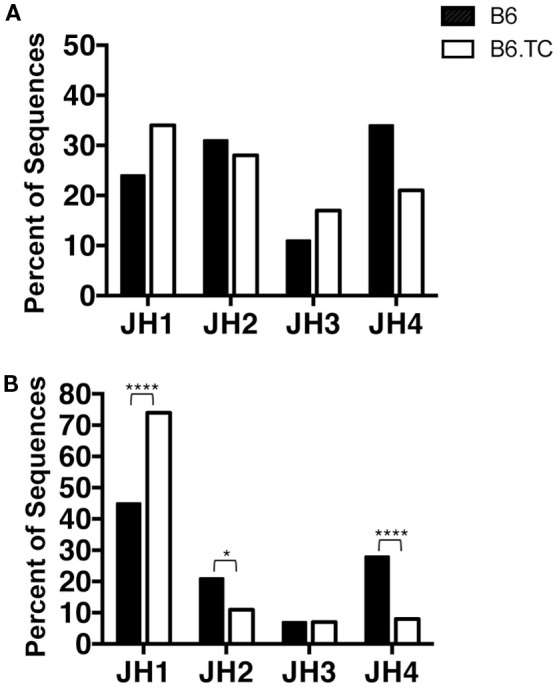
**J_H_ analysis of IgM from C57BL/6 and triple congenic mouse peritoneal B-1a cells**. Immunoglobulins were amplified by PCR from single-cell sorted peritoneal B-1a cells obtained from B6 and B6.TC mice and evaluated for the junction (J) segment heavy chain usage. The percent of cells (sequences) expressing the J segment usage is displayed. Chi-squared test was used to determine significance. **(A)** Analysis of sequences with only unique CDR-H3 regions (B6, *n* = 62; B6.TC *n* = 47). **(B)** Analysis of all sequences obtained, including the duplicate sequences (B6, *n* = 105; B6.TC *n* = 146).

We found numerous differences in V_H_–D_H_–J_H_ usage when analyzing all sequences, including the duplicate sequences (Figure 2B). V_H_1 and V_H_5 were expressed significantly less frequently by B6.TC B-1a cells (10 and 3%, respectively), as compared to B6 B-1a cells (55 and 9%, respectively) (*p* < 0.0001 and *p* = 0.0397, chi-square test). Conversely, V_H_11 and V_H_12 were utilized significantly more frequently by B6.TC B-1a cells (35 and 34%, respectively), as compared to B6 B-1a cells (11 and 10%, respectively) (*p* < 0.0001 and *p* < 0.0001, chi-square test). Among D_H_ gene segments, no difference was observed for the unique sequences between strains (Figure 3A). When all sequences were compared, DFL16.1 was expressed less frequently, and DSP was expressed more frequently by B6.TC B-1a cells (17 and 74%, respectively) as compared to B6 B-1a cells (54 and 32%, respectively) (*p* < 0.0001 and *p* < 0.0001, chi-square test) (Figure 3B). As for J_H_ segments, no difference was observed between strains among J_H_ gene segments in unique sequences (Figure 4A). However, when all sequences were compared, J_H_1 was expressed more frequently (74%) (*p* < 0.0001), and J_H_2 and J_H_4 were expressed less frequently (11 and 8%, respectively) (*p* = 0.0293 and *p* < 0.0001, respectively) by B6.TC B-1a cells as compared to B6 B-1a cells (45, 21, and 28%, respectively) (Figure 4B). Thus, distinct V_H_, D_H_, and J_H_ gene segment usage separated B6.TC from B6 peritoneal B-1a cells across all sequences.

### The B-1a Cell Repertoire Is Less Diverse in B6.TC Mice as Compared to B6 Mice

N-region addition provides diversity to the CDR-H3 region of antibodies *via* random insertion of nucleotides at the V–D and D–J junctions by the enzyme TdT. It is well-documented that peritoneal B-1a cells have limited N-addition due to the lack of TdT expression during fetal development (31). We analyzed N-addition at the D–J and V–D junctions and determined CDR3 length. No significant differences were found when analyzing sequences with only unique CDR-H3 regions (Table 2). In contrast, analysis of all sequences, including the duplicates, demonstrated significant differences between B-1a cells from B6.TC and B6 mice. We found that the number of N-additions at the D–J or V–D junctions of B6.TC B-1a cells was significantly less than B6 B-1a cells (*p* < 0.0001 and *p* = 0.0120, respectively) (Table 2). B6.TC B-1a cells were also found to contain significantly fewer N-additions when analyzing the sum of the two junctions as compared to B6 B-1a cells (*p* < 0.0001). We further examined CDR-H3 length and found, consistent with the differences in N-addition between B6.TC and B6 B-1a cells, the average CDR-H3 lengths differed significantly when analyzing all sequences (*p* = 0.0044), but did not differ when analyzing only sequences with unique CDR-H3 regions (Table 2). These results demonstrate that the B6.TC B-1a cell population expresses immunoglobulin that is less diverse due to fewer N-region additions as compared to the B6 peritoneal B-1a cell population.

**Table 2:** **N-region addition and CDR3 length analysis of IgM from C57BL/6 and triple congenic mouse peritoneal B-1a cells**.

	CDR-H3 length	V–D	D–J	Sum
B6	11.9 (±0.295)	1.74 (±0.265)	0.89 (±0.203)	2.63 (±0.338)
B6.TC	12.0 (±0.680)	1.57 (±0.322)	2.34 (±1.81)	3.92 (±1.80)

**With duplicate sequences**	**CDR-H3 length**	**V–D**	**D–J**	**Sum**

B6	12.5 (±0.208)	2.07 (±0.215)	0.533 (±0.127)	2.60 (±0.244)
B6.TC	11.7 (±0.224)	0.63 (±0.129)	0.329 (±0.148)	0.959 (±0.204)

*Immunoglobulins were amplified by PCR from single-cell sorted peritoneal B-1a cells obtained from B6 and B6.TC mice and evaluated for N-region additions and CDR3 lengths. The average number of N-additions at each junction or sum of the two junctions is displayed (± SEM)*.

**Figure 5:**
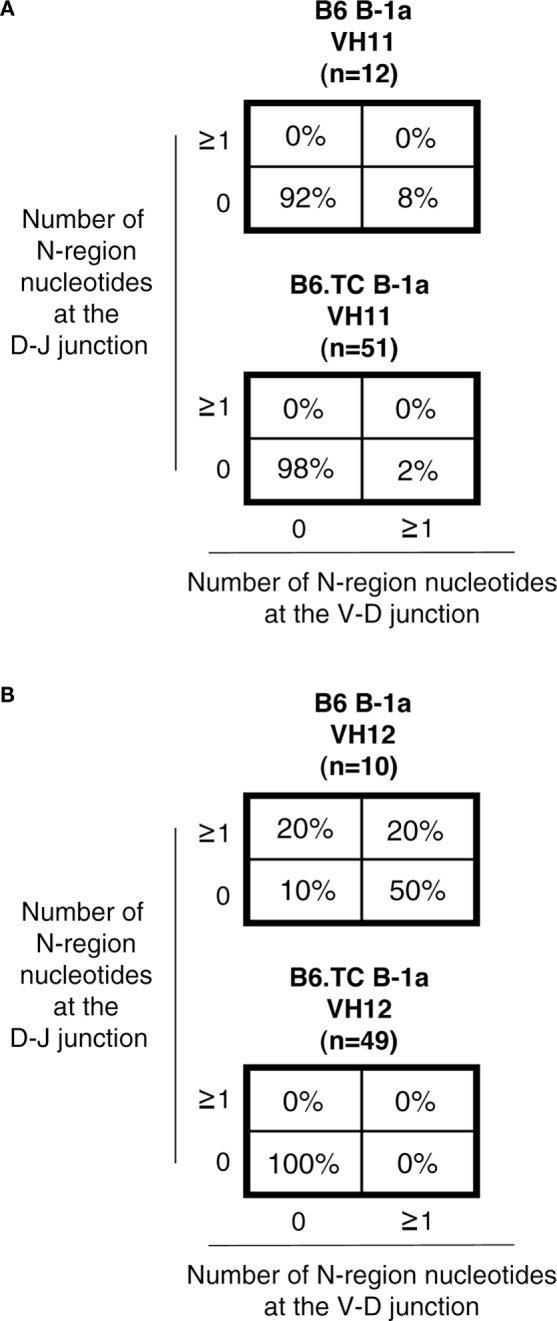
**N-region addition analysis of IgM from C57BL/6 and triple congenic mouse peritoneal B-1a cells utilizing V_H_11 and V_H_12**. Immunoglobulins were amplified by PCR from single-cell sorted peritoneal B-1a cells obtained from B6 and B6.TC mice and evaluated for N-region additions. The percent of sequences with 0 N-additions at both junctions, one or more N-additions at both junctions, 0 N-additions at V–D and one or more at D–J junctions, or 0 N-additions at D–J and one or more at V–D junctions is shown. **(A)** N-addition analysis of B-1a cells utilizing V_H_11 (B6 *n* = 12; B6.TC *n* = 51). **(B)** N-addition analysis of B-1a cells utilizing V_H_12 (B6 *n* = 10; B6.TC *n* = 49).

We then focused the analysis of N-addition on sequences utilizing V_H_11 and V_H_12, which are overrepresented in the B6.TC B-1a cells when considering all sequences, including the duplicate sequences. Interestingly, the number of B6.TC B-1a cell sequences lacking N-additions at both junctions in cells utilizing V_H_11 and V_H_12 (98 and 100%, respectively) was significantly different than B6 B-1a cell sequences (92 and 10%, respectively) (*p* = 0.0381 and *p* < 0.0001, respectively, by chi-square test), particularly with respect to V_H_12, although the number of B6 V_H_11/V_H_12 sequences was small (*n* = 12/*n* = 10). These results are summarized in Figure 5.

### CDR-H3s Are More Charged in B6.TC than B6 B-1a Cells

**Figure 6:**
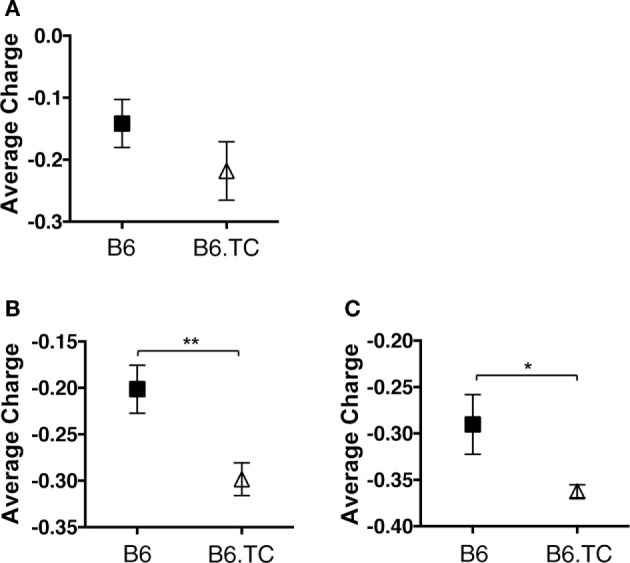
**Average charge of CDR-H3 loop region of IgM from C57BL/6 and triple congenic mouse peritoneal B-1a cells**. Immunoglobulins were amplified by PCR from single-cell sorted peritoneal B-1a cells obtained from B6 and B6.TC mice. IgM was amplified and sequenced as detailed in the Section “Materials and Methods.” The average charge of the CDR-H3 loop region of IgM is shown. **(A)** Analysis of sequences with only unique CDR-H3 regions (B6, *n* = 62; B6.TC *n* = 47). **(B)** Analysis of all sequences obtained, including the duplicate sequences (B6, *n* = 105; B6.TC *n* = 146). **(C)** Analysis of all VH11 and VH12 sequences, including the duplicate sequences (B6, *n* = 22; B6.TC *n* = 100).

Autoreactive antibodies, and in particular anti-dsDNA antibodies, are often enriched for charged amino acids in their CDR-H3 loop region (32–34). This region normally contains neutral, hydrophilic amino acids, which is due partly to usage of certain D_H_ and J_H_ sequences and use of the D reading frame I (32, 35). Amino acid changes within the loop region are also affected by N-region insertions (32). Upon evaluation of B6.TC B-1a cell CDR-H3 charge, we found that the average charge was increased in B6.TC B-1a cells (−0.213) over B6 B-1a cells (−0.142); however, this difference did not reach significance in the analysis of unique sequences (Figure 6A). When analyzing all sequences (including the duplicates), again the average charge of the CDR-H3 loop region was increased in B6.TC B-1a cells (−0.298) over B6 B-1a cells (−0.201) (*p* = 0.0015) (Figure 6B). Furthermore, comparing the average charge of the CDR-H3 loop region of B-1a cells utilizing V_H_11 and V_H_12 also demonstrated a greater charge in B6.TC B-1a cells (−0.362) than B6 B-1a cells (−0.290) (*p* = 0.0197) (Figure 6C). These results correlate with the differences observed in N-region addition between B6.TC and B6 B-1a cells utilizing V_H_11 and V_H_12.

## Discussion

Primary repertoire analysis of B-1a cells from 8-week-old B6.*Sle1.Sle2.Sle3* (B6.TC) lupus-prone mice demonstrated a large number of sequences that express identical CDR-H3 regions as compared to B-1a cells from healthy 8-week-old C57BL/6 (B6). This analysis demonstrates a significant increase in identical V_H_, D_H_, J_H_ usage in B6.TC mice. Although it is not possible to determine whether the duplicate sequences observed herein result from a single clonal expansion or from analysis of multiple cells with identical rearrangements, it has been well-documented over the years that B-1 cells have a limited repertoire (11, 14, 36–38), can undergo clonal expansion (39–42), and are self-replenishing (8). Therefore, these duplicate sequences are most likely due to expansion of single B-1a cells. Further analysis, including the duplicate sequences, reveals that the B6.TC B-1a cell repertoire displays early fetal/neonatal-like characteristics, which consists of an increase in use of J_H_1 [Figure 4B; Ref. (43)], few N-additions at both the V–D and D–J junctions, and a shorter average CDR-H3 length (Table 2). In addition, the B6.TC repertoire overused V_H_11 and V_H_12 as compared to B6 (Figures 1 and 2). Interestingly, V_H_11 and V_H_12 rearrangements are utilized almost exclusively by B-1a cells and target the cell membrane component PtC (19). Studies have shown V_H_11 in particular is a V_H_ gene utilized during fetal development but not during adult development (44, 45). More recently, Yang et al. have shown overuse of V_H_11 in the normal healthy peritoneal B-1a cell pool (38). Our results demonstrate the most common CDR3 in peritoneal B-1a cells from our normal healthy 2-month old B6 mice is ARRDYGSSYWYFDV (V_H_1-55, D_H_1-1, J_H_1). Examining Yang et al’s most common CDR3 in peritoneal B-1a cells from their normal healthy 2-month old B6 mice, it is ARFYYYGSSYAMDY, (V_H_1-55, D_H_1-1, J_H_4), which does not share the exact same CDR3 as ours but does share the same V_H_ and D_H_ region. Our second most common CDR3 sequences (two are tied for second place) are identical to Yang et al’s first and second most common CDR3 sequences ARFYYYGSSYAMDY and MRYGNYWYFDV (V_H_11-2, D2-8, J_H_1), respectively. The rank order of the sequences we identified is very similar to that of Yang et al. with only minor differences. Together, these results indicate that the B-1a cell repertoire in B6.TC mice reflects fetal rearrangements to a much greater extent than the B6 B-1a cell repertoire.

The mechanism for this selection toward fetal rearrangements in B6.TC mice is unknown; however, it can be speculated that the *Sle2c1* lupus susceptibility locus, which contains *Ckdn2c* and results in less p18 expression, could lead to a difference in expansion of B-1a cells with different BCR signaling requirements. It is possible the V_H_11 and V_H_12 specificities require a different level of BCR signaling, which the reduced level of p18 might provide, thereby allowing for increased proliferation of V_H_11 and V_H_12 expressing B-1a cells. In this view, *Ckdn2c* does not encode B-1a cell expansion on its own but does so in collaboration with BCR signals of requisite intensity. Our data suggest that such signals might be provided by self-antigen binding of PtC-specific immunoglobulins in preference to other elements of the B-1a cell repertoire.

Various studies have shown that B-1a cell-derived natural IgM provides protection against not only infection but also autoimmunity (9, 18, 46). The role of IgM in protection against autoimmunity was recently demonstrated in a mouse model lacking secretory IgM. This study demonstrated that natural IgM is required to control the accumulation of autoantibodies *via* its ability to regulate B cell development and selection (46). In mice lacking secretory IgM, the number of peritoneal B-1a cells was significantly decreased and of the B-1a cells present, there was little V_H_11 expression, which correlated with a lack of PtC-binding B-1a cells (46). These findings would seem to be at odds with previous studies demonstrating that mice with the *Sle2c1* lupus susceptibility locus have an expansion of peritoneal B-1a cells and yet they develop autoimmunity (28). Furthermore, results presented herein reveal that the B-1a cell repertoire in B6.TC mice is significantly more skewed toward V_H_11 and V_H_12 than control B6 mice (Figures 1 and 2). Together, these studies raise the question as to why the B6.TC mice are not protected against autoimmunity if they have an expansion of B-1a cells producing protective natural IgM. As demonstrated by Nguyen et al., selection of the B cell repertoire is affected by the presence of IgM (46). Herein, we demonstrate the expanded B-1a cells in mice carrying the *Sle2c1* lupus susceptibility locus are skewed toward a different specificity than control B6 mice. Together, these studies suggest that altering the pool of natural IgM disrupts the balance of antibodies, which enable selection of a healthy non-autoreactive repertoire. In other words, alteration of the natural IgM repertoire in the TC.B6 mice could then lead to selection of an autoreactive repertoire instead of a non-autoreactive repertoire, despite natural IgM being present. Furthermore, the role of B-1a cells in autoimmunity may not be limited to the antibodies they produce. B-1a cells have been shown to be potent antigen-presenting cells, which could also contribute to autoimmunity (7, 47, 48). In addition, B-1a cells have immunoregulatory functions through the secretion of IL-10 (49), ability to produce adenosine (50, 51), and ability to class switch in sites of inflammation (52).

The greater expansion/overuse of V_H_11 and V_H_12 might not help in the regulation of autoimmunity; however, it might afford increased protection from sepsis. It has been shown that the mice lacking secretory IgM are more susceptible to sepsis induced by cecal ligation and puncture (53). Interestingly, these mice could be rescued by injection of anti-PtC antibody, but not anti-PC antibody (53). Future studies could provide insight into whether the B6.TC lupus-prone mice might be more protected against bacterial sepsis. Such resistance to bacteria has been shown for mice with the *Sle3* lupus susceptibility locus (54); however, a role for B cells in such resistance has yet to be investigated.

The results presented herein demonstrate that the available B-1a cell repertoire present in 8-week-old B6.TC lupus-prone mice is more characteristic of an early fetal/neonatal B cell repertoire than that of B-1a cells from healthy aged-matched B6 mice. Thus, B6.TC-enhanced B-1a cell expansion is established early on and affects developing B-1a cells in a BCR-specific manner. Nonetheless, further analysis is required to determine the mechanism of B-1a cell expansion, and in particular, whether certain BCR specificities have a growth advantage in B6.TC mice. Overall, our results together with previous studies suggest the development of natural IgM that is protective against both bacterial infections and autoimmunity might require a balance of repertoire specificities. Previously published studies suggest this balance is greatly influenced by the IgM repertoire present (46). Further repertoire analyses of healthy and autoimmune models will help uncover factors that might affect this balance of protection against both infection and autoimmunity.

## Ethics Statement

Experiments using animals were conducted under approved Institutional Animal Care and Use Committee (IACUC) at the University of Florida.

## Author Contributions

Conceptualization and methodology, LM, NH, and TR; investigation, NH, LZ, and LM; writing – original draft, NH; ­writing – review and editing, LM, TR, and NH; funding acquisition, resources, and supervision, LM and TR.

## Conflict of Interest Statement

The authors declare that the research was conducted in the absence of any commercial or financial relationships that could be construed as a potential conflict of interest.
